# Efficacy of nasal high flow therapy on the coordination between breathing and swallowing of saliva during daytime nap in chronic obstructive pulmonary disease patients: A single center, randomized crossover controlled study

**DOI:** 10.1097/MD.0000000000021778

**Published:** 2020-08-21

**Authors:** Terumi Ayuse, Noriko Hisamatsu, Taiki Yamaguchi, Yosuke Takahashi, Yasushi Tamada, Shinji Kurata, Gaku Mishima, Max Pinkham, Stanislav Tatkov, Hideaki Takahata, Takao Ayuse

**Affiliations:** a Department of Special Care Dentistry; b Department of Dental Anesthesiology; c Fisher & Paykel Healthcare Ltd., Auckland, New Zealand; d Department of Rehabilitation Medicine, Nagasaki University Hospital; e Division of Clinical Physiology, Department of Translational Medical Sciences, Nagasaki University Graduate School of Biomedical Sciences, Nagasaki, Japan.

**Keywords:** chronic obstructive pulmonary disease, nasal high flow, saliva swallowing

## Abstract

**Background:**

There are some clinical reports on dysphagia in patients with chronic obstructive pulmonary disease (COPD); however, its pathophysiology remains largely unknown.

Changes in respiratory function occur in patients with COPD causing a decrease in tidal volume and an increase in respiratory rate (tachypnea). In addition, it leads to lack of coordination between respiration and swallowing.

A new treatment called nasal high flow (NHF) has been introduced for patients with COPD, replacing the traditional non-invasive ventilation (NIV) procedure. The NHF therapy involves inhalation of high flow of humidified air, which reduces respiratory effort in patients with COPD. Furthermore, NHF therapy facilitates swallowing of saliva even during respiratory management. A recent clinical study reported that high-flow nasal cannula oxygen therapy for 6 weeks improved the health-related quality of life and reduced hypercapnia in patients with stable COPD. Taken together, NHF therapy is gaining attention in the clinical management of patients with COPD.

Therefore, in this study, we aim to examine the efficacy of NHF therapy on the coordination between breathing and swallowing of saliva during daytime nap in patients with COPD.

**Methods/Design:**

This open-label, investigator-initiated, single center study will evaluate the efficacy of NHF therapy on the coordination between breathing and swallowing of saliva during the daytime nap in COPD patients with forced expiratory volume in 1 second (FEV_1_%) of <70% during treatment at the Nagasaki University Hospital Respiratory Rehabilitation Center. Evaluations will be performed during the 90 to 180 minute “daytime nap” in the measurement room of the hospital. The primary endpoint will be the rate of appearance of the expiratory phase after swallowing of saliva and the frequency of swallowing during the measurement period.

**Discussion:**

The purpose of this study is to obtain evidence regarding the utility of NHF as a potential therapeutic device for COPD patients to prevent aspiration of saliva during the sleep stage of daytime nap. The utility will be assessed by comparing the decrease in incidence rates of the expiratory phase after swallowing of saliva in the NHF device group and the control group, wherein this device was not used.

## 1 Introduction

There are some clinical reports on dysphagia in patients with chronic obstructive pulmonary disease (COPD)^[1]^; however, its pathophysiology remains largely unknown.^[2]^ Changes in respiratory function occurs in patients with COPD causing a decrease in tidal volume and an increase in respiratory rate (tachypnea). In addition, it leads to lack of coordination between respiration and swallowing, as well as a delay in the induction of the swallowing reflex. Studies have suggested that factors such as dyspnea associated with swallowing and lung hyperinflation may cause dysphagia.^[3]^

Coordinated movements of muscle groups related to breath-holding, such as the diaphragm and rectus abdominis, are important to control normal apnea during swallowing. The coordination between swallowing and respiratory movements may be impaired due to dysfunction of the breath-holding muscles and changes in lung capacity in COPD patients with a hunched posture. However, to date, the nature of the inhibition in coordination between swallowing and respiratory movements caused by changes in respiratory function in COPD patients has not been clarified.

Furthermore, aspiration of saliva during sleep as well as on awakening has been recognized as a major problem. The breathing pattern of an individual changes during the non-rapid eye movement (REM) and REM sleep periods, wherein the muscles of the pharynx relax, which may increase the risk of salivary aspiration compared with that during awakening.

The forward leaning posture with fixed upper limbs in patients with COPD increases the efficiency of the accessory respiratory muscles, namely the oblique and sternocleidomastoid muscles. The preliminary expiratory volume decreases as the degree of inspiration increases, comprising an increase in the index of kyphosis in order to compensate for chronic lung hyperinflation, exhalation restriction and weakness of the expiratory muscles.

Apnea is important to prevent aspiration during swallowing, and muscle functions related to breath-holding in the expiratory phase are considered to be easily affected by lung volume fractionation due to postural changes. We argue that, in order to control normal swallowing apnea, coordinated movements of muscle groups such as the diaphragm and rectus abdominis related to breath-holding are important; however, COPD is accompanied by changes in the dorsal-posterior posture of the patients. Therefore, we hypothesize that the coordination between swallowing and breathing movements is impaired due to dysfunction of the breath-holding muscles and the change in lung capacity.

A new treatment called nasal high flow (NHF) has been introduced for patients with COPD, replacing the traditional non-invasive ventilation (NIV) procedure. The NHF therapy involves inhalation of high flow of humidified air, which reduces respiratory effort and reduces respiratory function in patients with COPD.^[4–6]^ In addition, a study revealed that NHF therapy improved the respiratory work in patients during sedation with anesthetics.^[7]^ Furthermore, NHF therapy facilitated swallowing of saliva even during respiratory management.^[8,9]^ In a recent clinical study, Nagata et al^[10]^ reported that high-flow nasal cannula oxygen therapy for 6 weeks improved the health-related quality of life and reduced hypercapnia in patients with stable COPD. Nagata et al^[10]^ also conducted a multicenter randomized controlled trial to investigate the efficacy and safety of long-term high-flow nasal cannula oxygen therapy in stable COPD patients. Taken together, NHF therapy is gaining attention in the clinical management of patients with COPD.

**Figure 1:**
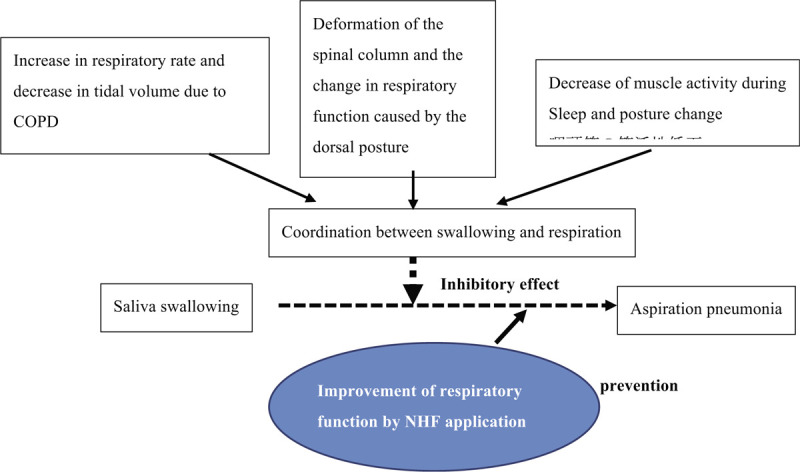
There are at least 3 factors affecting the coordination between swallowing and respiration during sleep stage. Increase in respiratory rate and decrease in tidal volume due to COPD. Deformation of the spinal column and the change in respiratory function caused by the dorsal posture. Decrease of muscle activity during sleep and posture change. Therefore, inhibitory effect on saliva swallowing by altered coordination between swallowing and respiration might occur silent aspiration during sleep. The application of nasal high flow with air could prevent “silent aspiration” by improving respiratory function during sleep stage in COPD patients. COPD = chronic obstructive pulmonary disease.

Therefore, in this study, we aim to examine the efficacy of NHF therapy on the coordination between breathing and swallowing of saliva during the daytime nap in patients with COPD. This is an original research to test the preventive ability of NHF therapy in aspiration of saliva occurring during the daytime nap in patients with COPD (Fig. 1).

## 2 Methods/design

### 2.1 Study design

The present study has been designed in accordance with the Standard Protocol Items: Recommendations for Interventional Trials and Consolidated Standard of Reporting Trials 2010 guidelines.^[11,12]^

**Figure 2:**
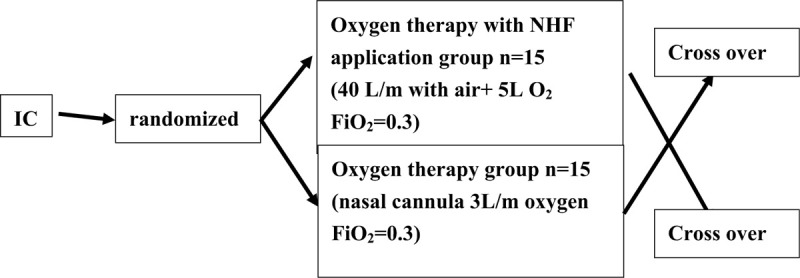
After obtaining informed consent, subjects who meet the registration requirements will be randomly assigned to either of the 2 groups. Fifty-percent of the patients will be randomly assigned to each group for this crossover study. The experimental group will be Oxygen therapy group with NHF application (40 L/m with air + 5 L O_2_ FiO_2_ = 0.3), control group will be Oxygen therapy group with nasal cannula (3 L/m oxygen FiO_2_ = 0.3).

This is an open-label, investigator-initiated, single center study on the efficacy of NHF therapy in COPD patients during the daytime nap as shown in Fig. 2.

The primary endpoint will be the occurrence rate of the expiratory phase after swallowing of saliva.

The Clinical Research Review Board of Nagasaki University has approved the study protocol. The study will be conducted at Nagasaki University Hospital in Japan. The study is registered in the jRCTs. The study will be conducted in accordance with the principles of the Declaration of Helsinki and the established best clinical practices of Japan. This is an exploratory study aimed at collecting information for conducting a verification study. Therefore, the number of cases will be set based on the feasibility in our hospital.

### 2.2 Participant recruitment

Participants will be recruited from the Nagasaki University Hospital, where the subsequent study will also be conducted. The treating clinical research coordinator (CRC) will explain the study to the recruited individuals, and an informed consent form will be signed by those who agree to participate. This is a randomized crossover study, and will comprise 2 groups of patients. Both groups will be tested during the daytime nap, and the NHF device will be used concomitantly in 1 group.

After obtaining consent, subjects who meet the registration requirements will be randomly assigned to either of the 2 groups. Fifty-percent of the patients will be randomly assigned to each group for this crossover study as shown in the figure below.

Fifteen patients with stable COPD, aged 40 years or older, who need oxygen therapy and show FEV_1_% of <70% will be recruited from the Nagasaki University Hospital Respiratory Rehabilitation Center. Patients with hypercapnia due to COPD (arterial blood carbon dioxide partial pressure [PaCO2 ≧ 45 Torr]) in the stable period, history of exacerbation of COPD (moderate or higher) in the past year, and those requiring home oxygen therapy will also be recruited. Patients with hypercapnia due to COPD (arterial blood carbon dioxide partial pressure [PaCO2 ≧ 45 Torr]) in the stable period, history of exacerbation of COPD (moderate or higher) in the past year, and those requiring home oxygen therapy will also be recruited.

### 2.3 Inclusion criteria

The proposed inclusion criteria are as follows: cases with moderate severity of COPD and moderate degree of air flow restriction according to GOLD guidelines (GOLD stages 2 and 3), adult patients aged 40 years or older at the time of obtaining consent. Any sex, outpatient cases, patients who, after receiving sufficient explanation before participation, have a thorough understanding of the study and provide written consent

### 2.4 Exclusion criteria

The proposed exclusion criteria are as follows: patients who cannot breathe through the nose, patients with a history of pneumothorax, patients with active malignant tumor(s), patients with acute illnesses (e.g., acute myocardial infarction), patients currently diagnosed with asthma (patients with a history of asthma but currently diagnosed with COPD will be eligible for inclusion.), patients diagnosed with obstructive sleep apnea syndrome in the past, and patients diagnosed with obstructive sleep apnea syndrome in the past, or those with strong clinical suspicion of the condition, patients who are already using the NHF device, patients who are using NIV at night, patients with severe disease of the kidney, liver, or circulatory system, patients deemed unsuitable for the study by the investigator

### 2.5 Study protocol

Evaluations will be performed during the 90 to 180 minute “daytime nap”^[13,14]^ in the measurement room of the Department of Anesthesiology and Biomedical Administration, Nagasaki University Hospital.

COPD patients, as an allocation factor, will be classified into 2 groups, a stage 2 group (80% ≧ FEV_1_ > 50%) and a stage 3 group (50% ≧ FEV_1_ > 30%), according to severity based on the standard FEV_1_.

#### 2.5.1 Assessment of swallowing

Swallowing function will be examined by 3 tests: revised drinking test (Modified Water Swallowing test (MWST)), repeated saliva swallowing test (RSST), and cervical chest auscultation method (CCA). In addition, the hoop index will be calculated in all patients to evaluate the degree of postural change. In order to assess the correlation between swallowing of saliva during breathing and respiration, the rate at which the expiratory phase appears after swallowing will be analyzed from the respiratory curve, and the time of swallowing apnea will be measured.

#### 2.5.2 Electromyography

An electromyogram (EMG) (manufactured by Bagnoli) will be used to measure muscle activity. The data measured by the EMG will be converted from an analog to digital signal (A/D conversion) at a sampling frequency of 1 kHz and loaded into a computer for analysis. Waveform analysis will be performed using myoelectric analysis software, and the bandpass filter will be set to 10 to 500 Hz. At the time of peak muscle activity, rest will be measured as 0 standard. The relative muscle activity will be calculated by normalizing the integrated value for 3 seconds during inspiration as 100% muscle activity

#### 2.5.3 Measurement of respiration

The coordination between swallowing and respiratory functions will be evaluated by a previously described method.^[13]^ Oxygen is administered through a nasal cannula in critically ill patients with COPD; therefore, respiration cannot be measured with a nasal flow sensor. Instead, an elastic band can be wrapped around the chest and abdomen, which can then be moved on the Impedance Plethysmograph, tracing the total movement of the chest and abdomen while swallowing. Apnea will be detected and lung parameters (lung volume) such as tidal volume, respiratory rate, and minute ventilation will be calculated. The starting time of swallowing will be recorded by the electromyogram. The starting time of swallowing will be recorded by the electromyogram. The transcutaneous CO_2_ concentration will be measured by attaching a TCO_2_ monitor (TCM5, manufactured by Radiometer, Japan, Tokyo) electrode to the earlobe, along with measurement of the transcutaneous oxygen saturation and pulse rate. The data will be stored in the internal memory of the device for subsequent retrieval and evaluation.

#### 2.5.4 Sleep cycle analysis

The sleep cycle during naptime will be continuously monitored using a wristwatch type evaluator (Xiaomi Mi Smart Band4 activity meter). Body movements and breathing will be comprehensively evaluated by the device and each phase of nap sleep (non-REM, REM) will be measured. At the same time, an electrode of an electroencephalogram amplifier (GMS, MWM20) will be attached to the frontal region of the head for detailed evaluation of the sleep cycle.

#### 2.5.5 Analysis of measurement data

Apnea of <1 second occurs during swallowing of saliva, which is a physiological characteristic, and functions to prevent aspiration by shifting to exhalation. However, aspiration may occur if a person swallows during the inspiratory phase instead of the inspiratory to expiratory transition phase. The analysis focuses on the interaction between swallowing function and respiration.

#### 2.5.6 Measurement of the index of kyphosis

Curvature of the spine from the 7th cervical vertebra to the 4th lumbar spinous process will be considered for quantitative evaluation of kyphosis using an 80 cm long flexible curved ruler (made by Shinwa) in an easy sitting position with no plantar contact. The shape will be traced on the paper as a straight line L (cm) connecting the seventh cervical and the fourth lumbar spinous processes of the curve. The distance from the straight line L to the apex of the curve will be H (cm), based on the formula by Milne et al.^[14]^ The ratio will be calculated as the index of kyphosis.

We will administer NHF (30–40 L/min of air and 5 L of oxygen [FiO_2_ = 0.3]) during nap sleep, in order to assess the immediate effect of the therapy on swallowing function in patients with COPD. Assess swallowing function. On the first measurement day, patients will be randomly allocated to either the high-flow nasal cannula oxygen therapy group or the no NHF therapy group with only oxygen (3 L oxygen [FiO_2_ = 0.3]) group (1:1). The second crossover measurement will be performed at least 1 week after the first with reversal of treatments in the 2 groups. In both groups, oxygen will be administered by a nasal cannula at a flow rate sufficient to cause an increase in PaO_2_ to over 60 mm Hg (oxygen saturation >90%, usually ≤3 L/min at rest). Patients with hypercapnia due to COPD (arterial blood carbon dioxide partial pressure [PaCO2 ≧ 45 Torr]) in the stable period, history of exacerbation of COPD (moderate or higher) in the past year, and those requiring home oxygen therapy (COPD GOLD stages 2 and 3) will be the target cases.

### 2.6 Adverse events

In this study, the NHF device will be secured onto the patient via a nasal cannula, and this could lead to discomfort. Moreover, an electrode for measurement of transcutaneous CO_2_ concentration will be attached to the anterior chest in both the NHF and non-NHF groups. This might cause temporary redness in patients with sensitive skin. Care will be taken to prevent undue harm to the patients in case of such adverse events.

### 2.7 Outcome

The primary endpoint will be the frequency of swallowing of saliva during the measurement period and the rate of appearance of the expiratory phase.

The secondary endpoints will be: time-weighted average value of TcPO_2_ (average partial pressure during the observation period), time-weighted average value of TcPCO_2_ (average partial pressure during the observation period), time-weighted average value of SpO_2_ (average oxygen saturation during the observation period), time-weighted average respiratory rate (average respiratory rate during the observation period).

### 2.8 Efficacy

We will evaluate the efficacy of the investigational device on a number of parameters, including the frequency of swallowing of saliva during the measurement period and the rate of appearance of the expiratory phase after swallowing.

Furthermore, we will evaluate other parameters such as time-weighted average value of TcPO_2_ (average partial pressure during the observation period), time-weighted average value of TcPCO_2_ (average partial pressure during the observation period), time-weighted average value of SpO_2_ (average oxygen saturation during the observation period), time-weighted average respiratory rate (average respiratory rate during the observation period).

### 2.9 Safety

The proposed safety evaluation indices of this clinical trial are as follows: adverse events are any undesired or unintended signs (including abnormal laboratory values, abnormal vital signs), symptoms, or illnesses that occur between the onset of use of the NHF device and the end of the last observation. The causal relationship between use of the device and occurrence of adverse events will not be of significance to the study. Pre-existing symptoms and diseases will be treated as complications and not adverse events. However, if the complications worsen after use of the NHF device, they will be treated as adverse events, and the day of confirmation of the deterioration will be the date of occurrence of the adverse event.

### 2.10 Data collection and management

The assignment and input tables to be used in this study will be created with the Research Electronic Data Capture (REDCap) software. The study will be conducted after the registered patients are allocated to groups and the data collected from the medical records are submitted to the researcher. A physician, co-doctor, and coworker will assign an ID to the medical records of each patient, which will be provided to the researcher. The Principal Investigator or Co-Researcher will approve the observation/inspection/evaluation data of each research subject immediately after confirming the content. The Principal Investigator and the Clinical Research Center Data Management staff will perform a visual and logical check of the data entered in the case report. The Principal Investigator or the Research Coordinator will be contacted in case of any discrepancies or queries regarding the data. The case will be fixed by performing data lock after resolving the issues and modifying the data (if indicated). The data management staff will be responsible for overseeing the process of correction of errors that arise after a case is locked. Proper conduct of the study will be monitored in accordance with the research plan.

### 2.11 Statistical analyses

Considering the exploratory nature of this study, we will estimate the incidence rate for each study parameter and use the information to calculate the necessary sample size for the verification study, in order to achieve statistical significance.

Specifically, the rate of appearance of the expiratory phase after swallowing of saliva and the frequency of swallowing during the measurement period in the NHF device and control groups will be calculated. The difference (ratio) between the 2 groups and the 95% confidence interval on both sides will be calculated. For other items, the average value and standard deviation for each group will be calculated manually.

## 3 Discussion

Aspiration pneumonia due to dysphagia, which has been reported to occur due to dysfunction of the breath-holding muscles, is often observed in COPD patients with postural changes. However, there is lack of clarity regarding the pathophysiology of the respiratory function affected by aspiration pneumonia caused by dysphagia in such patients. It has been reported that aspiration pneumonia due to dysphagia often occurs in patients with COPD, and dysphagia is observed in approximately 9% of the patients; however, its pathophysiology is unknown. Moreover, there is no established treatment for dysphagia in patients with COPD.^[3]^ Furthermore, spinal deformities, such as a dorsal posture, are often observed in such patients to compensate for respiratory disorders. Deterioration of respiratory function due to such postural changes can interfere with respiratory coordination during swallowing. Changes in respiratory function occur in patients with COPD causing a decrease in tidal volume and an increase in respiratory rate (tachypnea). In addition, it leads to lack of coordination between respiration and swallowing. It has been suggested that factors such as dyspnea associated with swallowing and lung hyperinflation may cause dysphagia.^[15]^ Coordinated movements of muscle groups related to breath-holding, such as the diaphragm and rectus abdominis, are important to control normal apnea during swallowing. However, patients with COPD may have a hunched posture resulting in lack of coordination between the breath-holding muscles. Coordination between swallowing and respiratory movements may be impaired due to muscle dysfunction, changes in lung capacity, and dyspnea.

However, to date, no study has clarified the effect of changes in respiratory function on the coordination of swallowing and respiratory movements. The goal of using a NHF device, during sleep stages of the daytime nap is to prevent silent salivary aspiration by improvement in the coordination between swallowing and respiration. The novel NHF treatment, which has replaced the traditional NIV procedure, involves inhalation of high flow of humidified air, reduces respiratory effort, and reduces respiratory function in patients with COPD.^[4–6]^ In addition, a study revealed that NHF also improved the ventilation during propofol sedation.^[7]^ Furthermore, NHF therapy facilitated swallowing of saliva even during respiratory management.^[8,9]^ Terzi et al^[16]^ reported the use of NIV for exacerbation of respiratory distress in patients with COPD, to secondarily reduce the frequency of swallowing during the inspiratory phase, and improve swallowing function. A study reported that aspiration is less likely to occur during NHF therapy than during NIV in both humans and animals.^[9]^

In a recent clinical study, Nagata et al^[10]^ reported that high-flow nasal cannula oxygen therapy for 6 weeks improved the health-related quality of life and reduced hypercapnia in patients with stable COPD. Taken together, NHF therapy is gaining attention in the clinical management of patients with COPD.

Therefore, in this study, we will examine the association between dysfunction in breath-holding in COPD patients with postural changes, such as hunchback, and the onset of respiratory and swallowing incoordination.

## Acknowledgments

The authors would like to thank the colleagues and staff at the Dental Anesthesiology Department of Nagasaki University Hospital for their support.

## Author contributions

Terumi Ayuse, Noriko Hisamatu, Gaku Mishima, Taiki Yamaguchi, Yosuke Takahashi, Yasushi Tamada, Sinji Kurata, Hideaki Takahata and Takao Ayuse are responsible for conceiving and designing the trial, planning data analysis, drafting the manuscript, and approving the final manuscript. Max Pinkham and Stanislav Tatkov are responsible for preparing and completing set up of the AIRVO device, including all equipments. Terumi Ayuse and Takao Ayuse will participate in data collection and are in charge of recruitment and treatment of patients. All authors will have access to the interim results as well as the capacity to discuss, revise, and approve the final manuscript.
